# Editorial: Biomarkers of Alzheimer’s Disease: The Present and the Future

**DOI:** 10.3389/fneur.2016.00158

**Published:** 2016-09-27

**Authors:** Sylvain Lehmann, Charlotte Elisabeth Teunissen

**Affiliations:** ^1^Biochimie – Protéomique Clinique, Montpellier University Hospital, Montpellier, France; ^2^Neurochemistry Lab and Biobank, Amsterdam Neuroscience, VU University Medical Center, Amsterdam, Netherlands

**Keywords:** CSF, blood, biomarkers, diagnosis, dementias, Alzheimer, validation, discovery

**The Editorial on the Research Topic**

**Biomarkers of Alzheimer’s Disease: The Present and the Future**

## Introduction

Alzheimer disease (AD) is a neurodegenerative disorder characterized by significant cognitive deficits, behavioral changes, sleep disorders, and loss of functional autonomy. The number of patients suffering from AD is growing rapidly as the population ages worldwide. AD represents the major cause of dementia and has become a major public health issue. Memory impairment is usually the earliest but also the core symptom of this disease. The diagnosis at a late stage is relatively easy. However, a delay in the diagnosis is damageable for the handling of patients in terms of optimal medical and social care. Moreover, early diagnosis is essential to start treatments, which are conceivably more effective at the prodromal stage. The actual interest of the scientific head-ways is therefore to optimize the diagnosis in prodromal stage of the disease and to propose personalized therapeutic solutions to individual patients. New revised AD diagnostic criteria include alteration of cerebrospinal fluid (CSF) biomarkers: a decrease in concentrations of amyloid peptides (Aβ42) and an increase in tau and phosphorylated-tau (p-tau) protein concentration. This recognition of CSF biological biomarkers for diagnosis of AD is a major step toward the “molecular” diagnosis and follow-up of the disease. However, many issues are still subject of debate and further developments in this field focus on:

– First of all, there is a large debate on the appropriate and optimal use of the current CSF amyloid and tau biomarkers that now face clinical implementation – when and how (alone or in combination) to use them: for AD screening, in routine, for early or atypical AD cases, etc.
– How to measure them in the most reliable way? A question that necessitates discussion on standard operating procedures, harmonization, quality control, establishment of reference material and methods, and comparison of analytical platforms (immunoassays, single and multiplex assays, and mass spectrometry).
– The use of biomarkers (and in particular Aβ42) for the stratification, the follow-up and to predict therapeutic responses of patients involved in therapeutic trials targeting AD pathology.
– How to improve the biological diagnosis of AD (in terms of sensitivity, specificity, or differentiation from other neurodegenerative disorders) with the help of new biomarkers? This major topic has two complementary aspects: the detection of additional Aβ and tau isoforms and the detection of completely new biomarkers (in particular from “omics” studies). Of note, there is not a week without the suggestion in the literature of a new putative biomarker for AD.
– Related to the above item, is the issue of a blood diagnosis of AD. As a matter of fact, validated AD biomarkers are currently detected only in the CSF, which limits their usage in screening or monitoring patients. The standardization of blood collection/assays for putative AD biomarkers is however far from the position of the CSF field, and it will need to be addressed specifically.
– The last but not the least important issue of current and new biomarkers is how they relate to the understanding of the pathophysiology of AD and to new therapeutic leads.

The articles of this research topic address most of the issues listed above. Chapter 1 addresses the use of the current biomarkers of AD, i.e., amyloid and tau related. Bros et al. focus on the analysis of tau using mass spectrometry as a way to detect and quantify specific tau isoforms, and Struyfs et al. describe the added value of pTau for differential diagnosis of dementia subtypes. Dorey et al. focus on the use of Aβ40 in a ratio to Aβ42, which is currently a hot topic in the field, demonstrating that it improves the interpretation of Aβ42 for the diagnosis of AD. Several diagnostic centers apply this ratio on a routine basis, while other centers are not yet using it. Will this paper provide the final verdict? The next two papers of this chapter focus on amyloid-related biomarkers, i.e., anti-amyloid antibodies and amyloid oligomers. The anti-amyloid antibodies could be useful tools to detect ARIA’s as reported in the contribution of DiFrancesco. ARIA’s are a threatening side effect of anti-amyloid therapies and thus important to detect early during treatment. Oligomers are promising biomarkers but suffer from their versatility: oligomers are unstable under physiological and laboratory conditions, which is problematical to control. A stable standard for quantification of oligomers in biomarker assays is needed and Kühbach et al. describe the performance of their newly developed standard. The last two papers of this chapter by Ritter and Blennow give an excellent view of the current state of the art on biomarker developments for clinical trials and dementia diagnosis.

Papers in the chapter 2 of the research topic address methodological and quality issues during analysis. The major part of the papers is the result of the activities of a strong international network within the 3-year duration of the joint programming for neurodegenerative diseases project, “BIOMARKAPD” (http://biomarkapd.org/). BIOMARKAPD aimed to standardize all aspects of the assessment of established and new fluid biomarkers for AD and Parkinson’s disease (PD). This was driven by the fact that large variation in biomarker measurements were reported between studies, both between and within centers. Such variations may be caused by pre-analytical, analytical, or assay-related factors. They seriously jeopardize the introduction of biomarkers in clinical routine and trials around the world. The BIOMARKAPD consortium has established a virtual and a central biobank that are open for use for biomarker research, as presented in the first article of this chapter by Reijs et al. Biobanks containing body fluids of well-characterized patient cohorts are an invaluable source for biomarker development and validation studies. The next two papers by Leitão et al. and Cicognola et al. address the influence of pre-analytical confounders on CSF biomarker results, including sample handling (centrifugation speed and temperature) and diurnal variation. Two of the important guidelines developed by the BIOMARKAPD project are also published in this chapter: Andreasson et al. present a practical guide for immunoassay validation. There was a lack of such a practical guide, though such a guideline would help uniform validation of novel biomarker tests for use in clinical practice. Moreover, this guideline increases the awareness of the importance of assay validation before implementation in research settings and clinical routine. The paper of Del Campo et al. next describes an optimal workflow for cost-effective biomarker immunoassay development. Researchers that perform biomarker identification studies, e.g., by proteomics, often need to develop novel immunoassays for their novel candidate biomarkers, with limited resources. Del Campo et al. describe a systematic and rational workflow to optimize the cost-effective development of such assays. Then, Galasko addresses the development of novel biomarker candidates using the omics approaches by giving a critical review of the progress and results obtained this far. Finally, the review by Schindler and Fagan emphasizes the important information of the pathology or trajectories of biomarkers that were obtained by studying biomarkers in families with dominant inherited Alzheimer’s diseases, which is an incredible precious cohort.

In the last two chapters, which focus on CSF and blood biomarkers, the different articles by Steen Jensen, Paquet, Lopez-Font, Zhao, Herbert, Inekci, Delaby, Fiandaca, and Baird propose new biomarkers such as plasma proteins of amyloid pathology, miRNA, cytokines, kinases, axonal proteins, lipids, and fragments of already known markers. These articles give valuable insight into the novel approaches which could result in the identification of biomarkers useful in the field of diagnosis or therapeutic trials.

In conclusion, the topic “Biomarkers of Alzheimer’s disease” is a very active topic that has a major importance for medical diagnosis, basic research, and therapeutics in the neurology and neuroscience field. CSF biomarkers are increasingly implemented in clinical routine to sustain the diagnosis of dementias knowing that there is a strong need for accurate, sensitive, and reliable biomarkers for AD. This will in fact help early diagnosis, targeted therapeutics, prognosis, and follow-up of patients. We are pleased that the current research topic gives a comprehensive state of the art of the use of the biomarkers, and projects good faith into the implementation of well-validated novel biomarkers for dementia in the future.

## Articles

### Chapter 1: State of the Art of CSF Amyloid Peptides and Tau Proteins Analysis

1. Antibody-free quantification of seven tau peptides in human CSF using targeted mass spectrometry,
2. Cerebrospinal Fluid P-Tau181P: Biomarker for Improved Differential Dementia Diagnosis,
3. Cerebrospinal Fluid Aβ40 Improves the Interpretation of Aβ42 Concentration for Diagnosing Alzheimer’s Disease,
4. Anti-Aβ autoantibodies in amyloid related imaging abnormalities (ARIA): candidate biomarker for immunotherapy in Alzheimer’s disease and cerebral amyloid angiopathy,
5. Application of an Amyloid Beta Oligomer Standard in the sFIDA Assay,
6. Fluid Biomarkers in Clinical Trials of Alzheimer’s Disease Therapeutics,
7. The past and the future of Alzheimer’s disease CSF biomarkers – a journey toward validated biochemical tests covering the whole spectrum of molecular events.

### Chapter 2: Preanalytics, Discovery and Validation of CSF Biomarkers

1. The Central Biobank and Virtual Biobank of BIOMARKAPD: A Resource for Studies on Neurodegenerative Diseases,
2. Chasing the Effects of Pre-Analytical Confounders – A Multicenter Study on CSF-AD Biomarkers,
3. Preanalytical Confounding Factors in the Analysis of Cerebrospinal Fluid Biomarkers for Alzheimer’s Disease: The Issue of Diurnal Variation,
4. A Practical Guide to Immunoassay Method Validation,
5. Facilitating the Validation of Novel Protein Biomarkers for Dementia: An Optimal Workflow for the Development of Sandwich Immunoassays,
6. Expanding the Repertoire of Biomarkers for Alzheimer’s Disease: Targeted and Non-targeted Approaches,
7. Autosomal Dominant Alzheimer Disease: A Unique Resource to Study CSF Biomarker Changes in Preclinical AD.

### Chapter 3: Novel CSF Biomarkers of Dementias

1. Biochemical Markers of Physical Exercise on Mild Cognitive Impairment and gDementia: Systematic Review and Perspectives,
2. Pro-Apoptotic Kinase Levels in Cerebrospinal Fluid as Potential Future Biomarkers in Alzheimer’s Disease,
3. Transmembrane Amyloid-Related Proteins in CSF as Potential Biomarkers for Alzheimer’s Disease,
4. microRNA-Based Biomarkers and the Diagnosis of Alzheimer’s Disease,
5. CSF Neurofilament Light Chain but not FLT3 Ligand Discriminates Parkinsonian Disorders.

### Chapter 4: Novel Blood Biomarkers of Dementias

1. The Potential of Pathological Protein Fragmentation in Blood-Based Biomarker Development for Dementia – With Emphasis on Alzheimer’s Disease
2. Central Nervous System and Peripheral Inflammatory Processes in Alzheimer’s Disease: Biomarker Profiling Approach
3. Plasma 24-metabolite Panel Predicts Preclinical Transition to Clinical Stages of Alzheimer’s Disease
4. Blood-Based Proteomic Biomarkers of Alzheimer’s Disease Pathology

## Author Contributions

The two authors contributed equally to editing the topic.

## Conflict of Interest Statement

The authors declare that the research was conducted in the absence of any commercial or financial relationships that could be construed as a potential conflict of interest. The reviewer NA and handling Editor declared their shared affiliation, and the handling Editor states that the process nevertheless met the standards of a fair and objective review.

